# Marine dissolved organic matter: a vast and unexplored molecular space

**DOI:** 10.1007/s00253-021-11489-3

**Published:** 2021-09-18

**Authors:** Teresa S. Catalá, Spencer Shorte, Thorsten Dittmar

**Affiliations:** 1 Institute for Chemistry and Biology of the Marine Environment (ICBM), University of Oldenburg, Oldenburg, Germany; 2 Institut Pasteur Paris, UTechS-PBI/Imagopole, 25-28 rue du Docteur Roux, 75015 Paris, France; 3 Institut Pasteur Korea, 16 Daewangpangyo-ro 712 beon-gil, Bundang-gu, Seongnam-si, Gyeonggido 13488 Republic of Korea; 4 Helmholtz Institute for Functional Marine Biodiversity (HIFMB) at the University of Oldenburg, Oldenburg, Germany

**Keywords:** Marine dissolved organic matter (DOM), Untargeted metabolomics, Ultrahigh-resolution mass spectrometry, High content screening, Cytological profiling

## Abstract

**Abstract:**

Marine dissolved organic matter (DOM) comprises a vast and unexplored molecular space. Most of it resided in the oceans for thousands of years. It is among the most diverse molecular mixtures known, consisting of millions of individual compounds. More than 1 Eg of this material exists on the planet. As such, it comprises a formidable source of natural products promising significant potential for new biotechnological purposes. Great emphasis has been placed on understanding the role of DOM in biogeochemical cycles and climate attenuation, its lifespan, interaction with microorganisms, as well as its molecular composition. Yet, probing DOM bioactivities is in its infancy, largely because it is technically challenging due to the chemical complexity of the material. It is of considerable interest to develop technologies capable to better discern DOM bioactivities. Modern screening technologies are opening new avenues allowing accelerated identification of bioactivities for small molecules from natural products. These methods diminish a priori the need for laborious chemical fractionation. We examine here the application of untargeted metabolomics and multiplexed high-throughput molecular-phenotypic screening techniques that are providing first insights on previously undetectable DOM bioactivities.

**Key points:**

• *Marine DOM is a vast, unexplored biotechnological resource.*

• *Untargeted bioscreening approaches are emerging for natural product screening.*

• *Perspectives for developing bioscreening platforms for marine DOM are discussed.*

## Introduction

Natural products (NPs) from plants have been used in various branches of traditional medicine for millennia (Chassagne et al. 2019) and, according to the World Health Organization (WHO), comprise primary health care therapy for ca. 80% of the population in developing countries (Farnsworth et al. 1985). Accordingly, for three decades, pharmaceutical companies have turned drug discovery efforts toward screening chemical libraries containing pure active compounds isolated from “medicinal” plants (Strebhardt and Ullrich 2008). As such the single “magic bullet” paradigm has dominated in the form of industrialized target-based drug-discovery screening campaigns trawling through ever larger chemical libraries (i.e., > 2 M compounds). However, such efforts have suffered high attrition rates, yielding limited success toward discovery of new first-in-class drugs (Chassagne et al. 2019). In this scenario, searching the potential of NPs is made difficult by technical constraints, for example the need to detect biological activities and isolate the active compounds responsible. Accordingly, novel screening strategies are actively sought (Horvath et al. 2016).

In comparison with chemical scaffolds from known drugs, NPs span a wider and different chemical space than synthetic derivatives (Feher and Schmidt 2002; Ganesan 2008; Grabowski and Schneider 2007), and still fewer than 20% of NP core structures and scaffolds are represented in commercial compound libraries (Hert et al. 2009). In this sense, NP chemical diversity, structural complexity, and their biological selectivity present both an opportunity and a technical challenge for the development of novel drugs (Atanasov et al. 2015; Clardy and Walsh 2004). It is striking that while most NPs come from plants, many of today’s most useful medicines come from bacterial sources (Bérdy 2005). Most bacterial metabolites are laboratory isolated, with only a small subset being understood at the level of the biological chemistry underlying their natural production (Davies 2006; Ueda 2021). Understanding how microbe interactions and micro-environment influence metabolite production and NP biosynthesis is an active area of research (Burgess et al. 1999; Patin et al. 2018; Traxler et al. 2013; Trischman et al. 2004), especially in the context of marine sediments (Tuttle et al. 2019). This is because marine NPs harbor the largest part of our planet’s natural biodiversity (Mora et al. 2011) and has resulted in a long tradition of searching for new bioactive compounds from marine sources (Blunt et al. 2014; Gerwick and Moore 2012).

Marine NPs are extracted from marine organisms, such as bacteria (Baran et al. 2011; Mansson et al. 2011; Shin et al. 2010; Wienhausen et al. 2017; Wietz et al. 2010), microalgae or macroalgae (La Barre et al. 2010; Parrot et al. 2019; Payo et al. 2011), fungi (Capon et al. 2003; Elnaggar et al. 2016; Höller et al. 2000; Kim et al. 2016; Klemke et al. 2004; Lang et al. 2007; Li et al. 2010; Luo et al. 2004), or animals (Alvarez et al. 2010; Connor and Gracey 2012; Ivanešivic et al. 2011; Karakash et al. 2009; Sarma et al. 2009; Schock et al. 2010; Soanes et al. 2011; Utermann et al. 2018). In recent years, the search for new marine NPs has strongly shifted from macroorganisms to microorganisms, whereby 57% of new marine NPs reported came from marine microbial sources (Carroll et al. 2019). In this context, marine dissolved organic matter (DOM) represents a new paradigm shift in the blue biotechnological field. Marine DOM consists a large degree of small organic acids with amphiphilic properties that can be extracted from seawater through adsorption onto hydrophobic resins and promises a yet unexploited potential for blue biotechnology (Catalá et al. 2020; Müller et al. 2020). However, studying the biotechnological potential of DOM requires a variety of technical challenges to be addressed, and chemometric analytical fractionation is necessary to identify and isolate specific bioactivities therein.

## Marine DOM: a plethora of chemicals

**Fig. 1:**
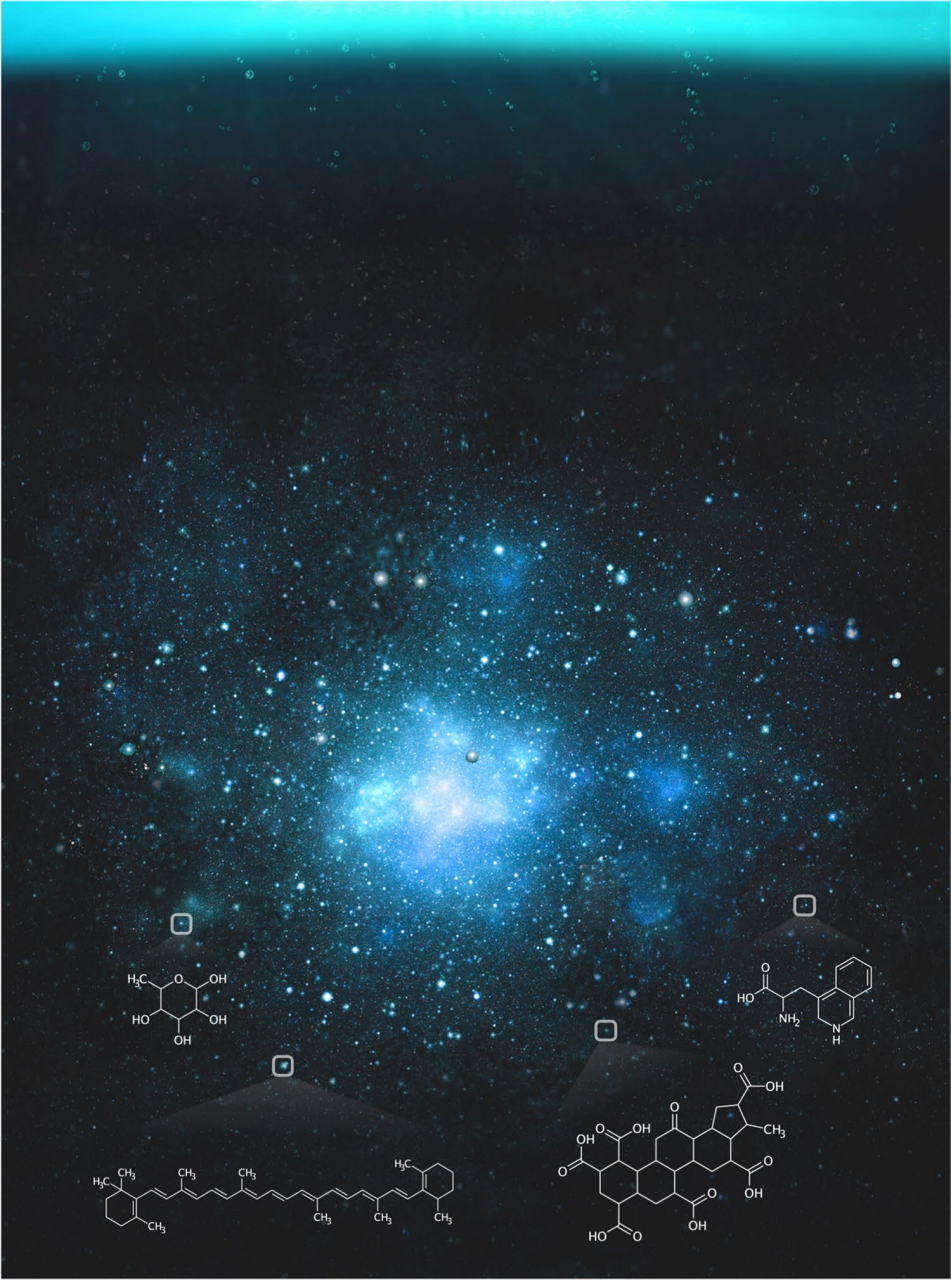
A molecular universe in the ocean. This illustration depicts the molecular complexity of marine DOM. Most of this pool is chemically unidentified (big nebulous), with only 2–3% of defined chemical structures (gray squares). The representative structures of marine DOM namely carotenoids, carboxylic-rich alicyclic molecules, and fucose were extracted from Arakawa et al. (2017), Hertkorn et al. (2006), and Repeta (2015), respectively

Marine DOM is one of the largest reservoirs of reduced organic carbon on the planet’s surface. The average liter of seawater contains < 1 mg of DOM, but considering the vast volume of the oceans, this adds up to a global reservoir exceeding 1 Eg of DOM (662 ± 32 Pg carbon; Hansell et al. 2009). As such, DOM contains a similar amount of carbon as atmospheric CO_2_ (860 Pg carbon; Friedlingstein et al. 2019) and holds > 200 times the carbon inventory of the total marine biomass (Hansell et al. 2009). DOM is continuously released by all organisms in the ocean while they live and upon death. In addition, water-soluble decomposition products from vascular plants are carried by rivers into the ocean. Most DOM quickly turns over by marine microorganisms, but a minor fraction turns over very slowly and has accumulated over several millennia to the observable pool of DOM. Marine DOM contains millions of different compounds of low molecular mass and, therefore, is unarguably one of the most complex chemical mixtures on Earth (Dittmar 2015), comprising extremely low concentrations of diverse chemical constituents (Arrieta et al. 2015; Zark et al. 2017). Many of the DOM compounds are alicyclic, organic acids with amphiphilic properties (Dittmar and Kattner 2003; Hertkorn et al. 2006; Zark et al. 2017), and are similar in structure regardless of the aquatic origin (Zark and Dittmar 2018). While these general structural features are known, the full structure of only a very minor fraction of the compounds residing in DOM is known (Dittmar and Stubbins 2014) (Fig. 1).

## Marine DOM: state-of-the-art analytical methods for bioactivity detection

Ironically, the chemical complexity that makes marine DOM so fascinating as a potentially rich source of bioactive molecules makes marine DOM so intractable to conventional chemoanalytical fractionation and characterization. Interest in natural extracts stems primarily from their potential as a source for new chemical scaffolds that might yield new *first-in-class* drugs. Thus, useful characterization of a natural extract requires the identification of constituent chemistry, and therein the associated corresponding bioactivities of interest, and ultimately, the isolation of the molecules involved, ideally with insight on their mechanism of action (MoA). Inasmuch as the complex mixtures of deep-sea DOM may contain millions of yet unknown chemical entities, it is a major challenge to efficiently distinguish the constituent bioactivity properties. However, recently, this challenge begins to yield in face of a convergence of technologies and methods underlying new analytical strategies for detection and deconvolution of bioactivities.

## Marine DOM: sample preparation and extraction

Marine DOM must first be isolated for biotechnological use and for molecular characterization. For these purposes, marine DOM must be separated from the seawater matrix that contains on average 35 g of inorganic salts, but less than 1 mg of DOM. Part of DOM can be extracted after acidification (pH 2) from seawater through adsorption onto hydrophobic resins (solid-phase extraction (SPE); Fu and Pocklington 1983). Currently, the most rapid and effective technique for this purpose uses a modified styrene divinylbenzene polymer type sorbent yielding recovery rates exceeding 60% of bulk DOM from marine environments (Dittmar et al. 2008; Green et al. 2014). SPE yields salt-free DOM samples that can be analyzed by high-resolution analytical technologies like mass spectrometry (Benner et al. 1992; Catalá et al. 2020). Compounds of higher molecular mass (> 1 kDa) or larger hydrodynamic diameter (> 1 nm) can be extracted from seawater via ultrafiltration. Ten to twenty percent of DOM falls into this high-molecular mass fraction. A promising, yet largely unexplored isolation technique is the combination of reversed osmosis and electrodialysis (RO/ED), which recovers up to 80% of DOM from oceanic waters (Koprivnjak et al. 2009; Vetter et al. 2007).

## Molecular characterization of marine DOM and chemometrics

Only few compounds can be separated with modern chromatographic techniques from the complex DOM mixture. As such, most efforts on molecular characterization of DOM rely on techniques that are capable to resolve molecular features of individual compounds or structures of complex mixtures. Chemometric data processing techniques have been developed to disentangle this complex information.

**Fig. 2:**
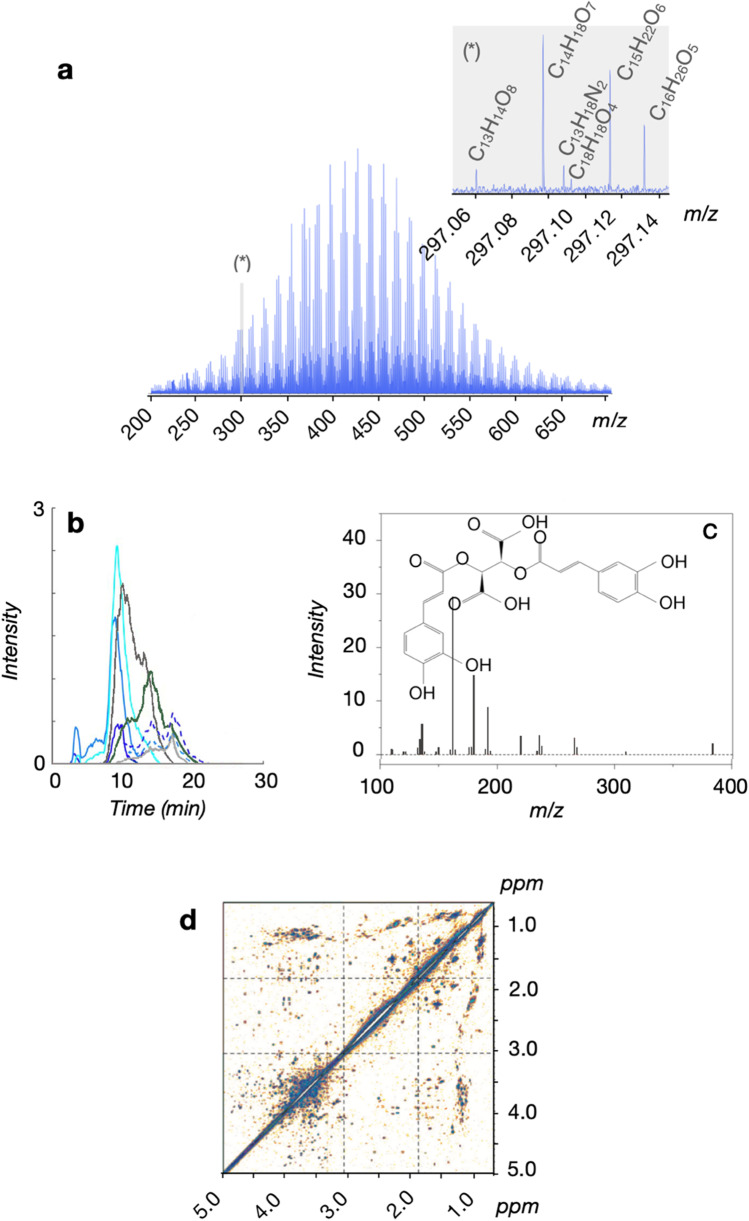
More than ten thousand molecular formulas and structural units have been identified in marine DOM. **a** A mass spectrum measured via ultrahigh-resolution mass spectrometry (15 T Fourier transform ion cyclotron resonance mass spectrometry, FT-ICR-MS) of the North Pacific Equatorial Intermediate Water with single asterisk indicating exemplary nominal mass (297 Da). **b** An HPLC–MS/MS scan of isomeric mixture peaks from Nordic Reservoir natural organic matter (NRNOM) reference material, depicting the inability to distinguish fragmentation patterns from individual isomers from a molecular mass (adapted from Hawkes et al. 2018). **c** An example of an assigned structure (C22H18O12) based on a riverine DOM MS/MS spectra and the METLIN database (adapted from Lu and Liu 2019). d The structural units identified by multidimensional nuclear magnetic resonance (NMR), in which each dot represents one structural feature (Hertkorn et al. 2012)

High-resolution mass spectrometry (HR-MS), coupled to bioinformatics tools, has gained popularity in non-targeted analyses (Ulrich et al. 2019; Hollender et al. 2017). Ultrahigh-resolution Fourier transform ion cyclotron resonance mass spectrometry (FT-ICR-MS) is a powerful tool specially for analyzing complex mixtures. Because of the high mass accuracy and resolution, this method detects thousands of molecular formulas in complex mixtures without iterative fractionation (Marshall et al. 1998; Nikolaev et al. 2016). In marine DOM, more than 10,000 molecular formulas and basic structural features have been identified with FT-ICR-MS (Hertkorn et al. 2013, 2016; Riedel and Dittmar 2014) (Fig. 2). FT-Orbitrap MS has become a widely available alternative HR-MS method in biogeochemical studies (Hawkes et al. 2016). It uses an electrostatic field rather than a magnetic field for separation of accumulated ions (Hu et al. 2005; Makarov 2000). Only via these HR-MS techniques, molecular formulas of individual compounds in highly complex mixtures such as DOM can be determined. However, HR-MS is unable to differentiate between structural isomers of a molecular formula (Zark et al. 2017; Hawkes et al. 2018).

The isomeric complexity within each molecular formula may be addressed via chromatographic separation prior to mass spectrometry or by fragmentation within the MS (Hawkes et al. 2018; Steen et al. 2020). Chromatography and other separation techniques allow the separation of DOM fractions, though only few individual compounds have been isolated from the complex mixture (Repeta 2015). Putative structural annotations and detection of specific molecular biomarkers in DOM can be achieved (McCarthy et al. 1998; Meyers-Schulte and Hedges 1986; Petras et al. 2017). Hawkes et al. (2018) used online high-performance liquid chromatography (HPLC) tandem HR-MS to explore the isomeric complexity of DOM. They concluded that fragmentation patterns from individual isomers from a molecular mass are indistinguishable, given a greater isomeric complexity that previously thought. They also concluded that isomeric complexity is a ubiquitous feature of DOM in all aquatic systems.

Liquid chromatography tandem mass spectrometry (LC–MS/MS) has become standard for compound identification in natural substances research, and recently, an untargeted approach has been developed for identifying putative library matches of specific molecular DOM compounds (Petras et al. 2017). LC can reduce sample complexity by separation molecules into fractions of different physical or chemical properties. In addition, MS/MS fragmentation provides additional information for structural elucidation and allows comparison with databanks, if available (Wang et al. 2016). When aligned LC–MS/MS spectra are used with molecular networking approaches (Aron et al. 2020; Petras et al. 2021), hundreds of unique molecular features can be classified at the compound level (Kujawinski et al. 2017; Petras et al. 2017). The online coupling of LC to HR-MS has also been introduced as an alternative to direct infusion HR-MS or to offline LC-HR-MS techniques, which have been the traditional biogeochemical approaches (Patriarca et al. 2018). Untargeted LC–MS/MS has been used to evaluate influences of microbial activities on the DOM composition. For instance, relevant lipids produced by diatoms were found in one of the first marine DOM studies to use this approach ( Kujawinski et al. 2017). Other studies proposed that putative amino acid–like and other compound classes were modified by bacterioplankton (Stephens et al. 2020), significant release of peptides by viral lysis of *Synechococcus* (Ma et al. 2018), and an association between elevated microbial community richness and DOM composition of temperate lake DOM (Muscarella et al. 2019). Furthermore, via such metabolite profiling, dozens of metabolites (e.g., guanine, glutamic acid, phenylalanine, and ectoine) have also been determined in a variety of marine samples and as intracellular metabolites within marine microorganisms (Johnson et al. 2020; Longnecker et al. 2018; Longnecker and Kujawinski 2020).

It is important to note that some compound classes of DOM escape mass spectrometric detection because they do not ionize efficiently (Patriarca et al. 2020). Another conceptual drawback of HR-MS via FT-ICR-MS or the Orbitrap technique is the long acquisition time. At highest resolution, a scan rate of 0.5 Hz or higher is required, which precludes coupling of fast separation techniques (such as GC) to HR-MS for the analysis of DOM. Time-of-flight mass spectrometers allow much faster scan rates, but largely because of their lower mass accuracy and resolution, application for the structural analysis of DOM has been scarcely developed (Lu et al. 2018, 2021).

Two-dimensional gas chromatography (GCxGC) coupled to MS was also used for the molecular-level identification of DOM compounds, such as terpenoids (Arakawa et al. 2017). This latter study proposed for the first time the abundance of isopropenoid structures in marine DOM. However, to separate the DOM components by modern GCxGC, chemical reduction from oxygen-containing functional groups into their respective hydrocarbon backbones or derivatization is required.

Nuclear magnetic resonance (NMR) spectroscopy is standard to determine molecular structures of DOM. NMR and fragmentation approaches combined with ultrahigh-resolution mass spectrometry have been applied to elucidate the structural diversity of DOM (Hertkorn et al. 2016; Kujawinski et al. 2016; Zark et al. 2017) (Fig. 2). NMR detects the resonance of NMR-active nuclei (i.e., ^13^C, ^1^H, ^15^ N, ^31^P) in the presence of a strong magnetic field (Steen et al. 2020). Chemical shifts from multiple nuclei are measured in two-dimensional (2D) NMR spectroscopy techniques, which allow resolution of overlapping peaks, verification of the interpretation of the chemical shifts, and identification of specific structures. ^1^H and ^13^C NMR spectroscopy revealed high structural diversity of DOM (Hertkorn et al. 2016) (Fig. 2) and resolved specific structural subunits, including carbohydrates and carboxy-rich alicyclic moieties, along with a minor amount of aromatic compounds, including *N*-heterocycles (Repeta 2015). The alliance of NMR and ultrahigh-resolution mass spectrometry to provide insights into the sources of refractory DOM components is on the rise (Abdulla et al. 2013; Aluwihare and Meador 2008; Hertkorn et al. 2006). Chemometric statistical methods allow combining NMR data with mass spectrometry data using multivariate statistics, for example to identify structural components and pathways of metabolic perturbations or to determine the biotransformation of metabolites on short timescales (Jaeger and Aspers 2014).

Complex datasets generated in targeted and untargeted metabolomics require novel tools for data analysis (Steen et al. 2020). Molecular networking and fragmentation tree algorithms have become key methods to visualize and annotate the chemical space in non-targeted mass spectrometry data. Metabolomics analysis software infrastructures, such as METLIN (Smith et al. 2005), mzCloud (https://www.mzcloud.org/), MetaboLights (Haug et al. 2020), or MetaboAnalyst (Chong et al. 2018), focus on search, annotation, and store of MS/MS spectra. However, neither of those allows free download of its reference library and enables searching a single MS/MS spectrum for identical or analogous MS/MS spectra in public data repositories (Wang et al. 2020). MZMine 2 (Pluskal et al. 2010) and XCMS (Smith et al. 2006) are processing methods that support both targeted and non-targeted analyses. SIRIUS 4 (Dührkop et al. 2019), ClassyFire (Feunang et al. 2016), and MetFrag (Ruttkies et al. 2019) also facilitate the ability to identify unknown compounds. Offering an assembly of all crucial data processing steps in combination with diagnostic tools for each critical step, the open access server-based tool ICBM-OCEAN offers an all-in-one tool applicable to any complex organic mixtures, including marine DOM (Merder et al. 2020).

Faced with the rapid growth in MS data availability and the deposition of untargeted MS data in the public domain (Haug et al. 2020; Perez-Riverol et al. 2019; Sud et al. 2016), the Global Natural Product Social Molecular Networking (GNPS) was developed to connect all public data (Wang et al. 2016). GNPS provides public dataset deposition and/or retrieval through the Mass Spectrometry Interactive Virtual Environment (MassIVE) data repository (Wang et al. 2016), the feature-based molecular networking (FBMN) as an analysis method (Nothias et al. 2020), and the web-based system MASST to search the public data repository part of the GNPS/MassIVE (Wang et al. 2020). Recently, the web-based GNPS Dashboard was also integrated to facilitate inspection, visualization, analysis, and sharing of private and public mass spectrometry data remotely (Aron et al. 2020; Petras et al. 2021). A first large-scale application of FBMN for the analysis of marine organic matter composition has been recently done (Petras et al. 2021). Other studies have used GNPS to get access to the complexity of DOM molecules at a more precise level of molecular annotation (Petras et al. 2017) or to create a molecular network and to search against GNPS spectral libraries (Stephens et al. 2020). These approaches consider the annotated features to be “putative” identifications that have not yet been verified by reference standards, but are based on spectral similarity to data from public or commercial libraries (Longnecker et al. 2015;Kujawinski et al. 2017).

Even though molecular networking, spectral and structural databases, statistical significance estimation, and in silico and community-based annotation criteria have significantly improved the annotation of MS/MS spectra (Horai et al. 2010; Wang et al. 2016; Watrous et al. 2012), the annotation of compounds that are not covered by spectral libraries remains as major challenges (Petras et al. 2021). This is particularly evident in marine DOM, as its molecular diversity exceeds that of any characterizable metabolome.

## Biological activity of marine DOM: phenotypic screening

Biological activity involves both molecular characterization and bioactivity assays. In face of this challenge, high-throughput molecular-phenotypic screening technologies combined with machine learning and statistical computational methods are emerging at the cutting edge. High-content screening (HCS) methods using image-based morphological analyses or “phenotypic screening” are showing unique promise as the technology tools for addressing the major challenges associated with screening natural product libraries and extracts, especially in the area of antibiotic discovery and infectious disease, notably *cytological profiling*.

Cytological profiling found its inspiration from next-generation concepts originally developed by early HCS studies using image-based phenotypic analysis in higher eukaryotic cells (Mayer et al. 1999; Mitchison 2005; Perlman et al. 2004). Specifically, milestone studies describing systematic multidimensional drug profiling by automated microscopy based on the premise that “…large sets of unbiased measurements might serve as high-dimensional cytological profiles analogous to transcriptional profiles” (Perlman et al. 2004). This experimental framework asserts that the cellular morphological phenotype may be considered albeit as a complex readout providing insight on the underlying biochemical, molecular, and ultimately genomic disposition of a response to perturbagen. This hypothesis encapsulates the postulate that the structural morphology of a cell visualized by means of optical microscopy comprises albeit convolved image features characteristic to specific epigenetic states. This profound yet intuitively self-evident idea has provoked considerable efforts to establish systematic means detect and quantify such characteristic features using a variety of innovative imaging methods. For a host of reasons, these efforts have emerged in the field of high-content screening for drug discovery where it has led to exciting developments aimed at enhancing phenotypic screening. For example, *Cell Painting* combines the powerful utility of subcellular organelle targeting fluorescent probes with machine learning to quantify literally thousands of image-based features per cell and, in turn, quantify specific morphological response patterns characteristic of chemogenomic perturbagens in higher eukaryotes (i.e., drugs, mutagenesis, disease states) (Bray et al. 2016, 2017; Bray and Carpenter 2018; Scheeder et al. 2018; Woehrmann et al. 2013). The utility of such measurements has even extended to providing insight on natural product libraries using single-cell phenotypic analysis in higher eukaryotic cells, for example HeLa cells (Kremb and Voolstra 2017), including “function first mode of action profiling” (Schulze et al. 2013), and “compound activity mapping” (Kurita et al. 2015).

Of special note are the *cytological profiling* studies reported by Nonejuie et al. (2013) and Woehrmann et al. (2013) because they pioneered the arguably more challenging imaging approach performed directly in bacterial cells rather than higher eukaryotic cells. Seminal work has demonstrated the value of cytological profiling and cluster-based analyses (e.g., principal component analysis (PCA), random forest (RF), etc.) in bacterial models to successfully identify candidate antibiotic molecules, and their corresponding mechanisms of action from natural product extracts and bacterial isolate libraries (Nonejuie et al. 2016; Wong et al. 2012). Indeed, these works have proven value demonstrating how phenotypic screens allow analyses of subtle submaximal doses of chemical perturbagens directly in bacteria based on the quantification of changes in bacterial cell features revealed by specific fluorescent stains for membranes, DNA, and membrane permeability. As reviewed recently, Genilloud (2019) observed: “The integration of this strategy in a multiparametric, high-content screening approach based on monitoring the lowest effective dose determining a phenotypic change has permitted the investigation of low potency hits from industrial collections and the identification of novel antibacterial compounds with differential modes of action at concentrations below the Minimum Inhibitory Concentration (MIC).”

Cytological profiling HCS methods are opening the way as a powerful means to investigate perturbagens displaying weak potency of inducing bacterial cell death or inhibiting bacterial growth under general screening conditions, making these methods ideal for identifying bioactivities in complex natural extracts and their fractions therein. As such, phenotypic strategies using cytological profiling could provide a new approach for detecting bioactivities in marine DOM.

## Combining data from multiple technologies opens new ways to analyze marine DOM bioactivities

Combining multiple data streams from different assays and/or screening technologies can provide powerful experimental recourse for describing biological states (sometimes referred to as “multiplexed” analyses). In this manner, image-based cytological profiling data combined with transcriptional expression can offer powerful complementarity for screening compound libraries. For example, Zoffmann et al. (2019) screened antibacterial activity of 1.5 million compounds from the Roche compound library against selected gram-negative pathogenic bacteria (*Acinetobacter baumannii*, *Escherichia coli*, *Klebsiella pneumoniae*, and *Pseudomonas aeruginosa*) using combined multiparametric HCS imaging and transcriptomics (gene expression). For each perturbagen, semi-automated image analysis provided a quantitative bacterial response profile: the “bacterial phenotypic fingerprint” (BPF) that when correlated with gene expression revealed prospective MoA. The manifold superiority of this approach stems from its capacity to detect subtle continuous effects compared with *binary* readouts common to conventional assays (i.e., bacterial growth or live/dead measurements). In this context, low-potency, weak antibacterial hits can, in fact, be exploited as leads to guide chemical structure activity relationship optimization toward antibiotic development.

It would be interesting to see how such multiplexed screening might help characterize marine DOM samples. Notably, an unresolved paradox is addressed in this review, namely why does bioactive DOM accumulate in the ocean? It was recently demonstrated that extreme substrate dilution limits the turnover of DOM in the ocean (Arrieta et al. 2015). According to this dilution theory, the individual components in DOM are simply too dilute in seawater to be reactive. Marine DOM is among the most complex molecular mixtures on Earth, and ten thousands of different molecular formulae and thousands of structural units (Fig. 2) have already been identified in this mixture to date (Hertkorn et al. 2006; Riedel and Dittmar 2014). Accordingly, the concentration of each compound is very low. The dilution theory implies that when sufficiently high concentrations are reached, most DOM becomes reactive. In fact, antioxidant potential and a clear microbial response were induced by increasing the marine DOM concentration (Arrieta et al. 2015; Catalá et al. 2020). As a classification tool, it could be potentially applied to screen for antibacterial compound leads, yielding simultaneously possible MoA, and hence might increase the chance to home in on previously unknown antibiotic (bioactive) chemical space.

Recently, a multiplexed screening strategy combined analysis using cytological profiling and metabolomics yielding bioactivity characterization termed “metabolic fingerprints of entire ecosystems” (Müller et al. 2020). First, untargeted ultrahigh-resolution mass spectrometry was used to capture the chemical space of 305 aquatic ecosystem samples collected across five continents (Europe, Africa, Australia, North America, and Antarctica). The “metabolome of entire ecosystems” (MeE) was defined as the entirety of small (< 1 kDa) molecules in these complex extracts. The MeE of all samples was then correlated according to screening results using a cell-based full-replication HIV-1 assay (EASY-HIT) (Kremb et al. 2010), uncovering samples capable of “strong” to “very potent” HIV-1 inhibition. Supervised machine learning extrapolated the highest statistical power for differentiation of anti-HIV activity, yielding ten molecular formula candidates of most potent anti-HIV characteristics. Following the assessment of antiviral activity, the complex extracts were subjected to a further comprehensive HCS approach: *Cell Painting*. Specifically, fluorescent dyes targeting 11 cellular structures (nucleus, actin, tubulin, mitochondria, whole cell, endoplasmic reticulum, lysosomes, membranes, NF-kB, caspase-9, p53) yielded 134 cellular measures and therein a characteristic cytological profile. Screening ecosystems containing at least one antiviral sample cytological profile were cross-referenced to cytological profiles obtained by the same method from a library of 720 bioactive reference compounds (Bray et al. 2017; Pennisi 2016). Orthogonal analyses using cytological profiling revealed HIV-inhibitory MeE samples in four major clusters. Remarkably, one of these was comprised from entirely unique characteristics indicating the presence of chemistry linked to a new MoA. Thus, performed on minimally processed complex mixtures, this study demonstrated how relatively low-cost multiplexed analyses can help mitigate risk associated with the decision to commit heavier resources (Schmitt et al. 1996, 1997; Woods et al. 2010, 2011), which are required for isolation of chemistry pursuant of worthwhile bioactivity.

## Concluding remarks and future directions

**Fig. 3:**
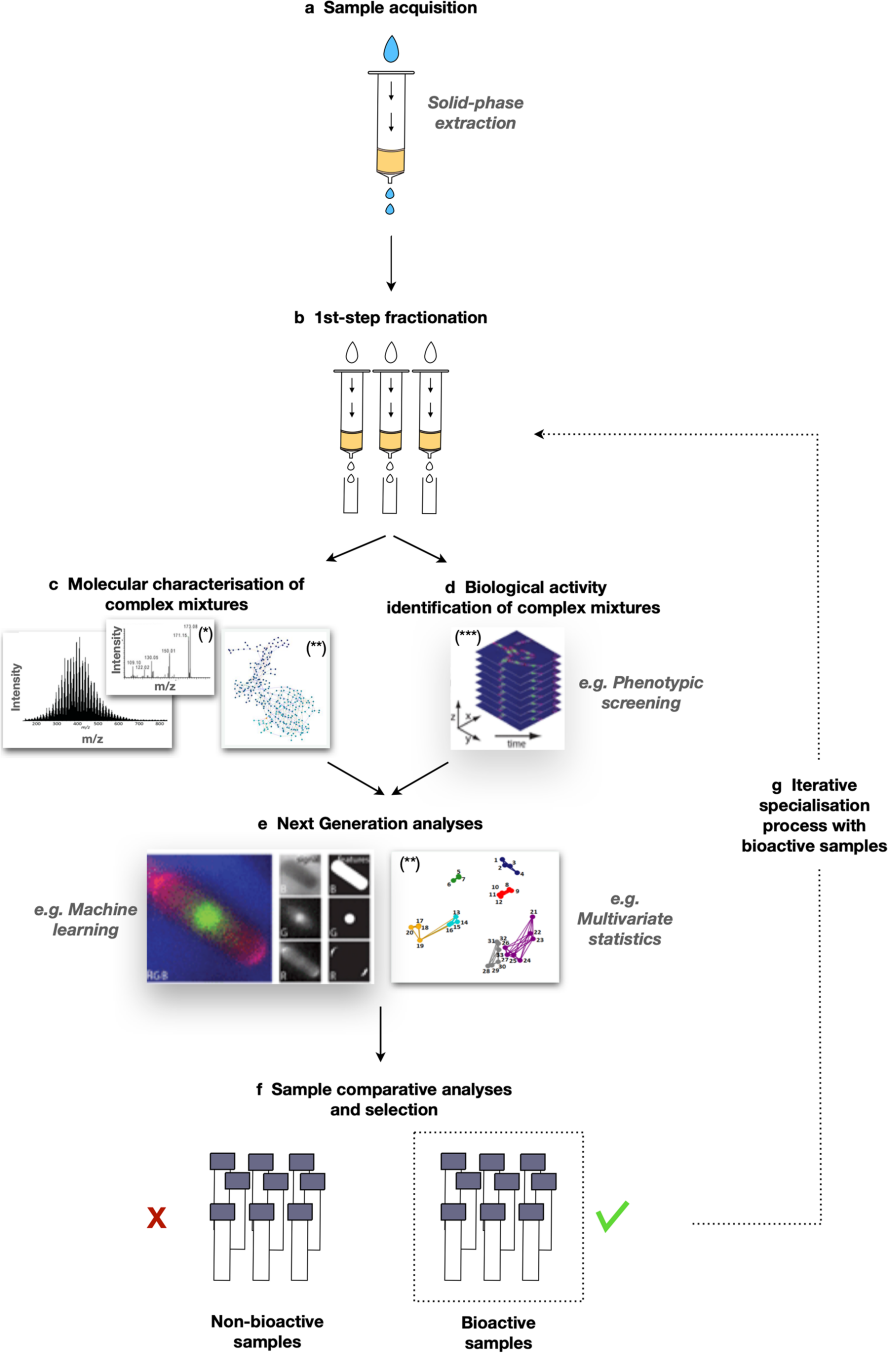
Emerging bioactivity screening paradigm targeting complex mixtures. **a** Sample acquisition via solid-phase extraction. **b** First-step fractionation. **c** Molecular characterization of complex mixtures via ultrahigh-resolution mass spectrometry (left), tandem mass spectrometry (middle), and molecular networks (right) to characterize chemomolecular fingerprints of natural extracts. **d** High-content phenotypic analyses: specific inhibition of cellular processes and/or bioactivity or function (e.g., cell growth/death; cell image–based profiling; gene expression/functional analysis (transcriptomics, proteomics, metabolomics), immune/inflammatory signal analyses, cytokines) and effect on infectious process (pathogen). **e** Next-generation analyses (e.g., multivariate statistical analyses, machine learning, neural networks, deep learning). **f** Iterative fraction-by-fraction comparative analyses and selection (positive, negative, and new activities, i.e., revealed or enhanced after fractionation). **g** Iterative specialization process with bioactive samples. Here, analyses can be developed beyond outright binary growth inhibition/cell death–type readouts and nuanced with more sensitive/sophisticated combinatorial and/or orthogonal readouts combining, for example, cytological characteristics, and/or more subtle molecular phenotypic readouts. Single asterisk, Petras et al. (2017); double asterisks, Merder et al. (2020); triple asterisks, Aulner et al. (2019)

Among the most compelling characteristics of DOM is the high chemical complexity that may provide a rich source of both previously unknown chemical scaffolds and new druggable targets. This same complexity raises challenges too; for example, chemical separation is made difficult, obfuscating isolation of specific molecules underlying specific MoA or biological activities of interest. Evidently, preliminary screening methods capable to assess bioactivity without chemical fractionation are a powerful asset to prospecting DOM samples. In this review, we have highlighted the emerging potential of both ultrahigh-resolution analytical techniques and phenotypic HCS methods that both appear to lend themselves as cutting-edge tools to help address these challenges. We propose interplaying technology workflows and integrating phenotypic HCS with ultrahigh-resolution MS, providing a guide to enhance conventional chemical separation and fractionation methods (Fig. 3).

Bioactivity research on DOM raises other intriguing questions, for example whether the bioactivity detected in complex organic mixtures is an effect of the specific activity of individual constituents, or an emergent *poly-pharmacological* property arising from the molecular diversity of the complex natural mixture. Multiplexed screening workflows like that shown in Fig. 3 using phenotypic cell-based assays are capable to provide empirical evidence of such emergent properties.

With the recent and general resurgent interest in natural extracts, bioactivities detected by empirical screening analyses, may not necessarily require chemical isolation in order to be legally subject to regulatory standardization. Distinct biological activities detected in samples collected from diverse ecosystems is burgeoning interest in DOM for a variety of biotechnological, pharmaceutical, and “cosmeceutical” applications (Catalá et al. 2020; Müller et al. 2020; Zhernov et al. 2016). Innovative combined technologies screening for bioactivities using untargeted biological models and chemical analysis are certainly helping dive deeper into characterization of the biotechnological potential of marine DOM.
